# Development of transdiagnostic clinical risk prediction models for 12-month onset and course of eating disorders among adolescents in the community

**DOI:** 10.1002/eat.23951

**Published:** 2023-04-13

**Authors:** Deborah Mitchison, Shirley B. Wang, Tracey Wade, Ann F. Haynos, Kay Bussey, Nora Trompeter, Alexandra Lonergan, Jack Tame MNeuroPsych, Phillipa Hay

**Affiliations:** 1 Eating Disorder and Body Image Network, Translational Health Research Institute, School of Medicine, Western Sydney University, Penrith, New South Wales, Australia; 2 Centre for Emotional Health, School of Psychological Sciences, Macquarie University, North Ryde, New South Wales, Australia; 3 Department of Psychology, Harvard University, Cambridge, Massachusetts, USA; 4 Flinders Institute for Mental Health and Wellbeing, Flinders University, Adelaide, South Australia, Australia; 5 Department of Psychology, Virginia Commonwealth University, Richmond, VA, USA; 6 Mental Health Services, South West Sydney Local Health District, Campbelltown, Australia

**Keywords:** eating disorder, illness course, onset, prediction model

## Abstract

**Objective:**

To develop and internally validate risk prediction models for adolescent onset and persistence of eating disorders.

**Methods:**

*N* = 963 Australian adolescents (11–19 years) in the EveryBODY Study cohort completed online surveys in 2018 and 2019. Models were built to predict 12-month risk of (1) onset, and (2) persistence of a DSM-5 eating disorder.

**Results:**

**Onset Model.**

Of the *n* = 687 adolescents without an eating disorder at baseline, 16.9% were identified with an eating disorder after 12 months. The prediction model was based on evidence-based risk factors for eating disorder onset available within the dataset (sex, body mass index percentile, strict weight loss dieting, history of bullying, psychological distress, weight/shape concerns). This model showed fair discriminative performance (mean AUC = .75). The most important factors were psychological distress, weight and shape concerns, and female sex.

**Diagnostic Persistence Model.**

Of the *n* = 276 adolescents with an eating disorder at baseline, 74.6% were identified as continuing to meet criteria for an eating disorder after 12 months. The prediction model for diagnostic persistence was based on available evidence-based risk factors for eating disorder persistence (purging, distress, social impairment). This model showed poor discriminative performance (mean AUC = .65). The most important factors were psychological distress and self-induced vomiting for weight control.

**Discussion:**

We found preliminary evidence for the utility of a parsimonious model for 12-month onset of an eating disorder among adolescents in the community. Future research should include additional evidence-based risk factors and validate models beyond the original sample.

**Public Significance:**

This study demonstrated the feasibility of developing parsimonious and accurate models for the prediction of future onset of an eating disorder among adolescents. The most important predictors in this model included psychological distress and weight and shape concerns. This study has laid the ground work for future research to build and test more accurate prediction models in diverse samples, prior to translation into a clinical tool for use in real world settings to aid decisions about referral to early intervention.

## 1 | INTRODUCTION

Despite eating disorders (EDs) affecting 21.0%–36.9% of adolescents, access to specialized treatment is extremely low (Hammerle et al., 2016; Micali et al., 2015; Mitchison et al., 2019), primarily due to a lack of detection and referral. This is true even for the better recognized conditions such as anorexia nervosa and bulimia nervosa, in which only 10%–27% of those affected are estimated to have accessed ED specific healthcare (Fatt et al., 2019; Swanson et al., 2011). Established screening instruments and methods have good sensitivity and specificity for detecting a *current* ED (Hill et al., 2010; Maguen et al., 2018; Solmi et al., 2015); however, their ability to predict *future* outcomes, such as onset and persistence of an ED, is unclear. Key risk factors, such as weight and shape concerns and other sociodemographic (e.g., female gender) and clinical features (e.g., strict dieting), have well-known prognostic value in predicting variance in ED onset (Bakalar et al., 2015; Culbert et al., 2015; Day, Bussey, Trompeter, & Mitchison, 2021; de Portela Santana et al., 2012; Ghaderi, 2001; Glashouwer et al., 2019; Jacobi et al., 2004; Keel & Forney, 2013; Lie et al., 2019; Mazzeo & Bulik, 2009; N. Micali, 2005; Stice, 2002; Vall & Wade, 2015). However, to date these have not been integrated into algorithmically-driven screening procedures that would optimize precise prediction of these key outcomes, and facilitate early intervention.

In other areas of psychiatry, prognostic model research (also known as “clinical risk prediction modeling”) has been applied to predict outcomes such as likelihood to develop a new onset of psychosis over a 5-year period in the UK National Health Service with good accuracy (Fusar-Poli et al., 2018, 2019; for a summary of model performance indicators and their interpretation, see Supporting Information File 1). Prognostic modeling is a specific methodology (Steyerberg et al., 2013) with recommended reporting guidelines (the TRIPOD Statement [Collins et al., 2015]) that makes use of the evidence base from risk factor and early modeling (Fairburn et al., 2005) research to build models that provide an overall risk score for each individual. The translational outcome of such research, online risk calculators that can be used by clinicians and consumers to guide help-seeking, allocation to early intervention, and treatment decisions (as part of evidence-based practice alongside clinician expertise and client preferences), would be a considerable advancement to the ED field. The value to these models is capturing people before they develop an ED, so that we can make better use of early interventions, which have been shown to result in better outcomes than treatment as usual (Richards et al., 2022). Another ED outcome for which accurate prediction would be of high value is the likelihood of persistence of an ED among those already affected in the community who are yet to receive treatment. For instance, predicting likelihood of ED persistence could assist in the primary care setting regarding decisions about the level of intervention to recommend following screening. This could matched to level of risk for persistence from a watch-and-wait approach, to guided-self-help, to referral to ED specific treatment—ultimately improving the efficiency of EDs healthcare.

The development of prognostic models using methodologies that align with the TRIPOD Statement (Collins et al., 2015) has only recently commenced for outcomes related to EDs. These models include the prediction of future persistence of ED diagnosis based on a wide range of clinical and sociodemographic factors (Haynos, Wang et al., 2021), current ED diagnosis based on internet activity (Sadeh-Sharvit et al., 2020), and response to ED treatment based on pretreatment clinical variables (Espel-Huynh et al., 2021). In a study by Haynos, Wang et al. (2021), risk prediction models were developed for ED persistence and presence of specific symptoms after 1 and 2 years using a pre-existing dataset of 320 females with an established ED. The models predicting the future presence of specific ED symptoms showed “fair” to “good” accuracy (mean “area under the receiver operating characteristic curve”—AUC range: 0.71–0.89) and better than the models predicting persistence of EDs (mean AUC range: 0.61–0.62, “poor” accuracy). Models built utilizing a machine learning method (ML; elastic net) also outperformed models built using traditional logistic regression. It is important to note here that the extent to which these models performed well in part depended on the large number of risk factors (33 in total) included, as more information in models improves precision. However, it is also true that more complex models pose a challenge for eventual translation into real world settings as they involve greater administrative burden (Steyerberg et al., 2013). Nevertheless, these first studies demonstrate promise for the application of ML methods to the development of prediction models for ED outcomes, including using self-report data.

The aims of the current study were to develop the first prognostic models for ED onset (Aim 1) and persistence (Aim 2) in the community. Data were from adolescents (the peak age of ED onset) in the Every-BODY cohort who completed self-report surveys over 12 months. A previous study with this cohort observed 18.2% new onset cases (inclusive of subthreshold disorders) over a 12 month period (Prnjak et al., 2021). With the goal to develop models that may eventually be translated into clinical practice, only the best available predictors were selected for the models, based on evidence from systematic reviews and meta-analyses. While a body of studies suggest ML is comparable to logistic regression for building risk models (e.g., Espel-Huynh et al., 2021), emerging evidence suggests ML is superior when models are more complex (e.g., more predictors; Haynos, Wang et al., 2021) or when accuracy of prediction is a priority (Wang, 2021). Hence, we tested both approaches in order to further inform methodological decision-making in this area. The study was conducted in alignment with the reporting guidelines outlined in the TRIPOD (Transparent Reporting of a Multivariable Prediction Model for Individual Prognosis or Diagnosis) Statement (Collins et al., 2015).

## 2 | MATERIALS AND METHODS

### 2.1| Data source

The data were from Wave 1 and Wave 2 (i.e., the first and second follow-up surveys) of the EveryBODY Cohort, a representative population-based self-report study of EDs among Australian adolescents. These waves were chosen as they were the most inclusive of the predictors and outcomes of interest.

### 2.2 | Study population

Participants were those who attended one of eight secondary schools in New South Wales, Australia, and participated in the online surveys in both Wave 1 (2018) and Wave 2 (2019) of the EveryBODY study. Four public and four private schools were included, and the sample were demographically representative of adolescents in the State of New South Wales. Surveys were administered at schools between May and December based on the school’s preference for roll-out. Details of the recruitment procedures and the EveryBODY cohort have been published previously (Prnjak et al., 2021; Trompeter et al., 2018). The retention rate from Wave 1 to Wave 2 was 61.6%. When comparing those who did versus did not participate in Wave 2, there was no difference in gender distribution, however, Wave 2 completers were slightly younger (14.62 vs. 15.08 years on average; *p* < .001,${\text{η}}_{\text{p}}^{\text{ 2}}\;=\;0.021$), had a slightly lower BMI percentile (50.92 vs. 53.77 on average; *p* = 0.014,${\text{η}}_{\text{p}}^{\text{ 2}}\;=\;0.002$), and were slightly more likely to have been born in Australia (84.8% vs. 80.4%; *p* = 0.45, V = 0.056). These effect sizes were all small, and largely explained by the fact that students who were in their final year of school at Wave 1 were very difficult to recruit for the Wave 2 survey when they were no longer in school. For Aim 1, we developed models with data from participants who did not meet criteria for an ED at Wave 1 and who had complete data for diagnosis at Wave 2 (N = 687). For Aim 2, we developed models with data from participants who did meet criteria for an ED at Wave 1 and had complete data for diagnosis at Wave 2 available (N = 276). Approval for the study was granted by the Macquarie University Human Research Ethics Committee and the New South Wales Department of Education. All participants assented to the study and their parents/guardians provided passive informed consent.

### 2.3 | Study measures

#### 2.3.1 | Outcomes

The two outcomes were (1) ED onset (meeting criteria for a probable ED at Wave 2, but not Wave 1), and (2) ED persistence (meeting criteria for a probable ED at Wave 1 and Wave 2). As described previously, diagnoses were determined based on self-report responses to a range of standardized measures (including the Eating Disorder Examination Questionnaire, Fairburn & Beglin, 2008; K10 Psychological Distress Scale, Kessler et al., 2002; and Pediatric Quality of Life Scale, Varni et al., 2003) and specific questions designed by the investigators to assess specific diagnostic criteria (see Supporting Information File 2 for full information; Mitchison et al., 2019).

#### 2.3.2 | Predictors

We selected predictors based on replicated evidence of their prognostic value for ED onset and persistence, respectively, as summarized in meta-analyses, systematic and expert narrative reviews (Bakalar et al., 2015; Culbert et al., 2015; Day, Bussey, Trompeter, & Mitchison, 2021; de Portela Santana et al., 2012; Ghaderi, 2001; Glashouwer et al., 2019; Jacobi et al., 2004; Keel & Forney, 2013; Lie et al., 2019; Mazzeo & Bulik, 2009; N. Micali, 2005; Stice, 2002; Vall & Wade, 2015). As recommended, continuous variables were not dichotomized (Fusar-Poli et al., 2018; Fusar-Poli et al., 2019).

Predictors for the models in Aim 1 (ED onset) included binary sex (Weissman, 2019), body mass index (kg/m^2^; BMI) percentile adjusted for child age and sex (Bakalar et al., 2015; Stice, 2002), days of weight loss dieting over the preceding one month (Bakalar et al., 2015; Stice, 2002), history of being a victim of bullying at school (Bakalar et al., 2015; Day, Bussey, Trompeter, & Mitchison, 2021; Lie et al., 2019), frequency of psychological distress over the preceding one month (Bakalar et al., 2015; Culbert et al., 2015; Keel & Forney, 2013; Stice, 2002), and severity of weight/shape concerns over the preceding one month (Bakalar et al., 2015; Glashouwer et al., 2019; Keel & Forney, 2013). Predictors for the models in Aim 2 (ED persistence) came primarily from two reviews based on anorexia nervosa and bulimia nervosa (H.-C. Steinhausen, 2002; H. Steinhausen & Weber, 2009) and included frequency of purging, psychological distress, and perceived social functioning over the past month.

*BMI percentile* was based on self-reported height and weight and adjusted for the adolescent’s sex and age. Self-report of height and weight data in adolescents has been recommended when direct measurement is impractical, because even though there is a slight tendency for underestimation of weight, self-report and directly measured data remain highly correlated (Kee et al., 2017; Sherry et al., 2007).

*Weight loss dieting* was measured using an author-derived question, “*Over the past 28 days (4 weeks) how many days have you been on a very strict weight loss diet?*” and participants indicated their response by free text. Evidence of the convergent validity of this item include the significant association between scores on the item and scores on measures of other disordered eating behaviors (Aouad et al., 2019); (Pursey et al., 2020), and weight-related bullying (Day, Bussey, Trompeter, Hay, et al., 2021).

*History of bullying* was assessed with the author-derived question “*Have you ever been bullied at school?*” and participants indicated their response by selecting “no” or “yes.” This is a similar question to that employed in other adolescent population-based surveys. However, in the present study the question was prefaced with a standard definition of bullying, as recommended by bullying research experts to improve construct validity (Day, Bussey, Trompeter, Hay, et al., 2021; Day, Bussey, Trompeter, & Mitchison, 2021).

*Psychological distress* was measured using the *Kessler Psychological Distress Scale* (K-10) (Kessler et al., 2002), a well-validated questionnaire frequently used in population studies, which assesses symptoms of anxiety and depression over the last 4 weeks on 10 Likert-type questions. Scores range from 10 to 50, with higher scores indicative of greater distress. Internal consistency in the Aim 1 sample was ω = 0.89 and in the Aim 2 sample was ω = 0.93.

*Weight and shape concerns* were assessed using the combined items of the *Weight Concern* and *Shape Concern* subscales of the *ED Examination Questionnaire (EDE-Q)* (Christopher G Fairburn & Beglin, 2008). This combined scale has been well-validated in Australian adolescents (J. Mond et al., 2014) and consists of 12 Likert-type items assessing dissatisfaction, preoccupation and overvaluation with weight/shape over the past 4 weeks. Scores are averaged across the items, ranging from 0 to 6, with higher scores indicative of greater weight/shape concerns. Internal consistency in the Aim 1 sample was ω = 0.92.

*Purging* was assessed using two separate items in the *EDE-Q* (Christopher G Fairburn & Beglin, 2008) that asked participants to indicate the frequency of self-induced vomiting and laxative use for weight control purposes over the previous 28 days. These items have been used previously in Australian adult (Mitchison et al., 2012, 2014) and adolescent (Fatt et al., 2019; Trompeter et al., 2020) population studies.

*Social functioning* was assessed using the *Social Functioning* subscale of the *Pediatric Quality of Life Scale (PedsQL) short form* (Varni et al., 2003). The well-validated subscale consists of 3 Likert-type items that assess frequency of social functioning impairment over the previous 28 days. Scores range from 0 to 100, with higher scores indicating lower levels of social functioning impairment. Internal consistency in the Aim 2 sample was ω = 0.85.

### 2.4 | Statistical analyses

Preliminary analyses included computation of descriptive statistics for each sample. Univariate logistic regression analyses were conducted to examine the unadjusted relationships between each candidate predictor and the outcomes and are presented in Supporting Information File 3.

#### 2.4.1 | Modeling approach

Analyses were performed in R version 3.6.1 via caret (Kuhn, 2021) and glmnet (Friedman et al., 2010) packages. To predict EDs onset and persistence, we conducted elastic net regularized logistic regressions. We chose the elastic net algorithm given its well-established accuracy and robustness, its ability to maintain clinical interpretability compared to less transparent ML algorithms (e.g., random forests, neural networks), and its validity in prediction ED risk models in a prior paper (Haynos, Wang et al., 2021). For each outcome (i.e., in Aim 1 and Aim 2), we also compared predictive accuracy of elastic net models with nonregularized logistic regression models, as has been done previously (Haynos, Wang et al., 2021). As per the additional aim of this study, the ML versus logistic regression models were compared based on the model performance metrics listed below (see Supporting Information File for further information on interpretation of these metrics).

All models included participants with complete data available for outcomes (i.e., ED diagnostic information at Wave 2). Regarding missing data for predictors, there was 5.28% missingness for Aim 1 (predicting probable ED onset) and 0.87% missingness for Aim 2 (predicting persistence of probable ED). We used K-nearest neighbor imputation during data preprocessing for all models. Given that, our data were imbalanced (with fewer individuals meeting criteria for a probable ED at Wave 2 than those not meeting criteria for a probable ED), we used upsampling to improve the balance across classes in all models (Kuhn & Johnson, 2013).

To obtain metrics of predictive accuracy, we followed recommendations (Kuhn & Johnson, 2013), and used 10-fold cross-validation with three repetitions to select the optimal λ (shrinkage) and α (mixing) parameters for each elastic net model; we also used the same cross-validation procedure for logistic regression models. Repeating the training and testing process in this way can provide more reasonable estimates of model performance for future datasets than splitting a sample into a single training and testing set, particularly for smaller samples. Finally, we evaluated variable importance for all models with the varImp() function in caret.

#### 2.4.2 | Model performance

A standard metric for examining a model’s performance is the area under the receiver operating characteristic curve (AUC, which measures area under a curve with 1—specificity on the x-axis and sensitivity on the y-axis). An AUC of 0.5 indicates chance-level predictive accuracy and an AUC of 1.0 indicates perfect classification (50–0.59 = extremely poor; 0.60–0.69 = poor; 0.70–0.79 = fair; 0.80–0.89 = good; 0.90–1.00 = excellent). We also evaluated several other classification metrics, including the average cross-validation estimates of: area under the precision-recall curve (AUPRC), accuracy, positive predictive value (PPV), sensitivity, specificity, and Brier score For more information on each of these metrics and their interpretation, see Supporting Information File 1.

## 3 | RESULTS

### 3.1 | Sample characteristics

Demographic and clinical characteristics of the subsamples included in this study are presented in Table 1. A description of the demographic characteristics of the full EveryBODY cohort, which closely approximate the general adolescent population in New South Wales, have been published elsewhere (Mitchison et al., 2022).

#### 3.1.1 | Aim 1: ED onset

There were *n* = 687 participants without an ED at Wave 1, comprising the sample for Aim 1. Just over half of this sample was male, mostly born in Australia (84%), with an average age of 14.4 years, and with an average BMI percentile within the Center for Disease Control (CDC) definition of “healthy.” Wave 1 disordered eating was low in the sample with only 1.2% scoring above the cut-off for extreme weight and shape concerns (scoring ≥4 on the EDE-Q combined Weight and Shape Concerns scale; Mond et al., 2014), and at-least-weekly fasting, objective binge eating, self-induced vomiting, and laxative use reported by only 4.5%, 5.4%, 0.6% and 0.9% of participants, respectively. At Wave 2, 116 (16.9%, 95% CI: 14.3–19.9%) of these participants had developed a probable ED. This rate was expected based on a previous study (Prnjak et al., 2021) using earlier waves of this cohort, and when considering the high rates of prevalence (19%–37%) for the full spectrum of EDs globally (Hammerle et al., 2016; Micali et al., 2015).

#### 3.1.2 | Aim 2: ED persistence

There were *n* = 276 participants with a probable ED at Wave 1, comprising the sample for Aim 2. Three quarters of this sample was female, mostly born in Australia (84%), with an average age of 15 years, and an average BMI percentile that was within the CDC definition of “healthy.” The probable EDs that were observed in this sample included *n* = 11 with anorexia nervosa, *n* = 66 with bulimia nervosa, *n* = 21 with binge ED, *n* = 39 with atypical anorexia nervosa, *n* = 35 with subthreshold bulimia nervosa, *n* = 11 with subthreshold binge ED, *n* = 37 with purging disorder *n* = 81 with night eating syndrome, and *n* = 12 with unspecified feeding/ED. Only 13.8% reported having ever had seen a health professional for a problem with their body image. At Wave 2, when assessed 1 year later, 206 (74.6%, 95% CI: 69.2–79.4%) participants in this sample continued to meet criteria for a probable ED. Access to treatment for a body image problem remained low, increased only marginally to 14.9% by Wave 2.

### 3.2 | Model performance

#### 3.2.1 | Aim 1: Predicting EDs onset

**FIGURE 1:**
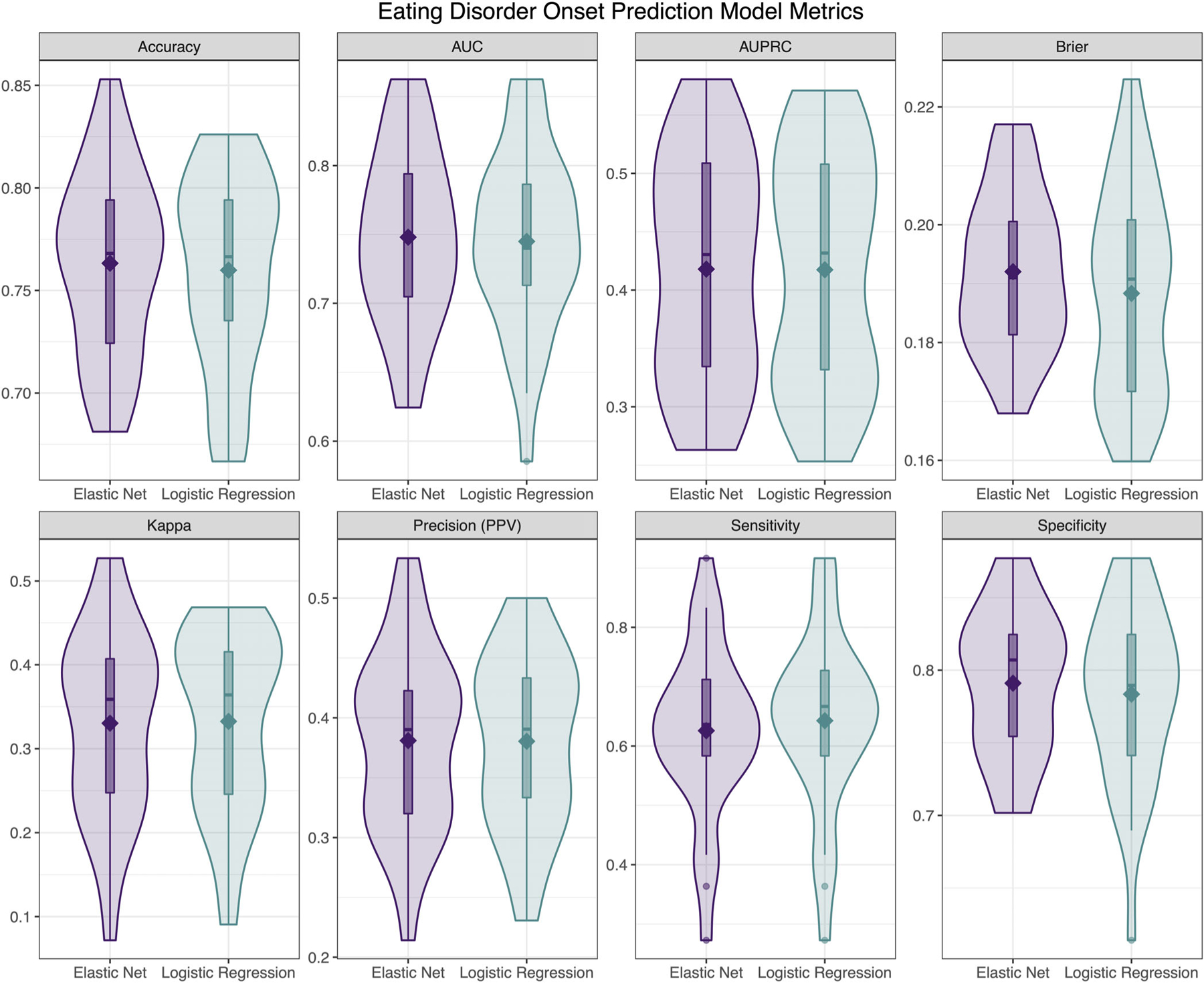
Descriptive visualizations of the distribution of cross-validated model performance metrics of elastic net and logistic regression models predicting eating disorders onset at Wave 2 based on Wave 1 data (N = 687). AUC, area under the receiver operating characteristic curve; AUPRC, area under the precision-recall curve.

Model performance metrics for the logistic regression and elastic net models predicting probable ED onset are presented in Figure 1. The logistic regression model provided fair AUC (mean cross-validated AUC = 0.75), with poor AUPRC (*M* = 0.42), fair accuracy (*M* = 0.76), fair PPV (*M* = 0.38), poor sensitivity (*M* = 0.64), fair specificity (*M* = 0.78), and fair Brier score (*M* = 0.19). The most important variable in the logistic regression model was psychological distress (*β* = 8.01), followed by weight/shape concerns (*β* = 5.81), female sex (*β* = 3.50), being a bully victim (*β* = 2.98), BMI percentile (*β* = 1.82), and strict weight loss dieting (*β* = 0.16).

The elastic net model showed nearly identical performance to the logistic regression model, with fair AUC (*M* = 0.75), poor AUPRC (*M* = 0.42), fair accuracy (*M* = 0.76), fair PPV (*M* = 0.38), poor sensitivity (*M* = 0.63), fair specificity (*M* = 0.79), and fair Brier score (*M* = 0.19). The most important variable was also psychological distress (*β* = 0.54), followed by weight/shape concerns (*β* = 0.37), female sex (*β* = 0.22), being a bully victim (*β* = 0.14), BMI percentile (*β* = 0.02), and strict weight loss dieting (*β* < 0.001).

#### 3.2.2 | Aim 2: Predicting EDs persistence

**FIGURE 2:**
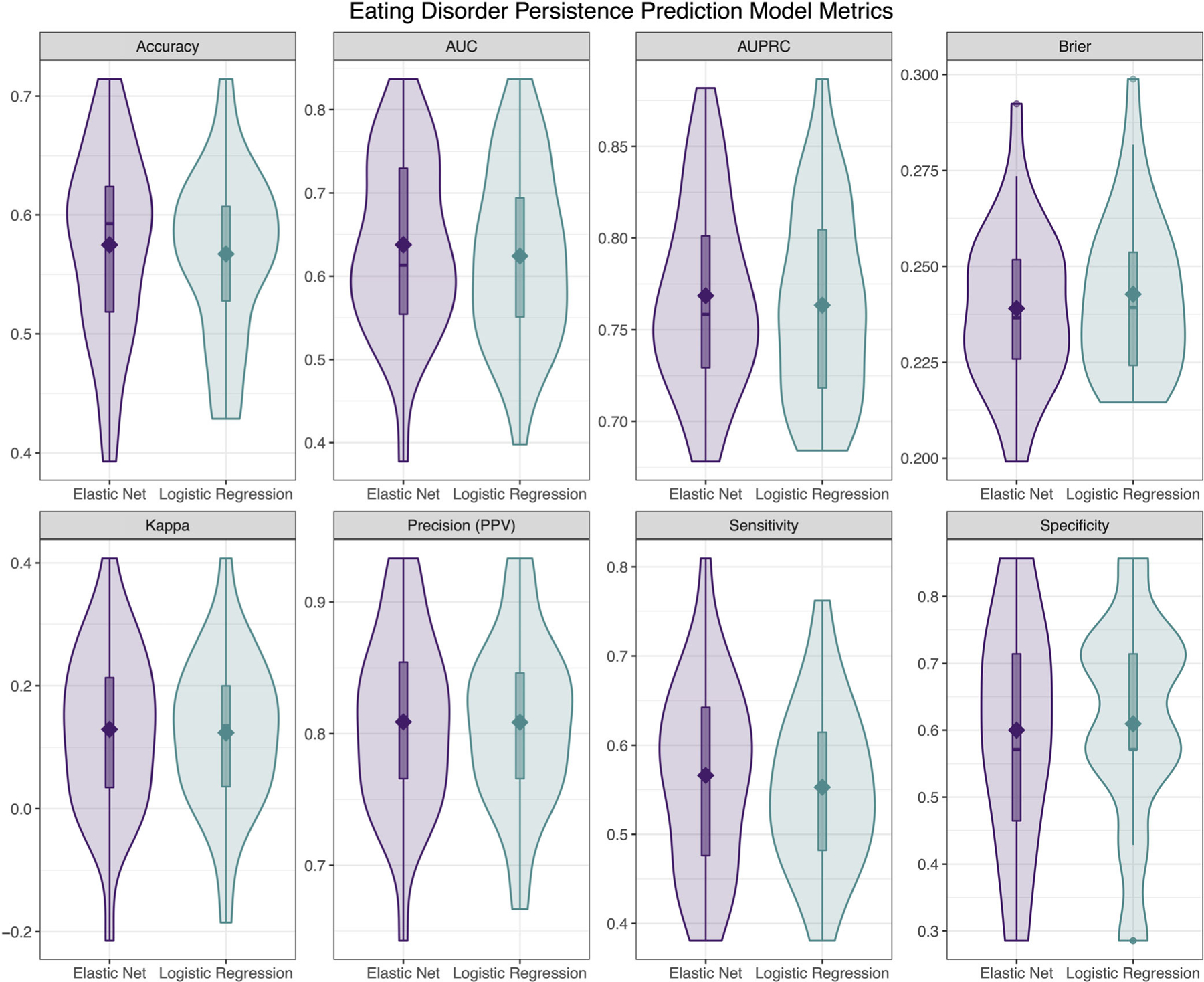
Descriptive visualizations of the distribution of cross-validated model performance metrics of elastic net and logistic regression models predicting eating disorders persistence at Wave 2 based on Wave 1 data (N = 276). AUC, area under the receiver operating characteristic curve; AUPRC, area under the precision-recall curve.

Model performance metrics for the logistic regression and elastic net models predicting persistence of a probable ED are presented in Figure 2. The logistic regression model provided poor AUC (*M* = 0.62), with fair AUPRC (*M* = 0.76), poor accuracy (*M* = 0.57), good PPV (*M* = 0.81), poor sensitivity (*M* = 0.55), fair specificity (*M* = 0.61), and poor Brier score (*M* = 0.24). The most important variable in the logistic regression model was psychological distress (*β* = 3.07), followed by self-induced vomiting (*β* = 1.45), social functioning (*β* = 1.27), and laxative use (*β* = 0.31).

The elastic net model showed nearly identical performance to the logistic regression model, with poor AUC (*M* = 0.64), fair AUPRC (*M* = 0.77), poor accuracy (*M* = 0.57), good PPV (*M* = 0.81), poor sensitivity (*M* = 0.57), fair specificity (*M* = 0.60), and poor Brier score (*M* = 0.24). The most important variable was also psychological distress (*β* = 0.34), followed by self-induced vomiting (*β* = 0.19), social functioning (*β* = 0.14), and laxative use (*β* = 0.03).

## 4 | DISCUSSION

This study presents, to our knowledge, the first general population mixed gender prognostic models for probable ED onset and persistence among adolescents. A focus was on model parsimony to enhance translatability for eventual use in the real world. Our model predicting probable ED onset performed well, accurately discriminating between future cases versus noncases 75% of the time, and positively identifying 64% of future cases (sensitivity) and 79% of future noncases (specificity). However, the model predicting persistence of a probable ED was poor, with accurate discrimination between future cases versus noncases closer to chance levels (64%). This is likely due to there being a lack of relevant predictors included in this model, an important focus for future research.

The performance metrics for the model predicting probable ED onset were in the range observed for established and implemented prognostic models within other areas of health and medicine (e.g., cancer and heart disease [Fusar-Poli et al., 2015]). Although the values for discrimination, sensitivity and specificity were nominally labeled as fair, poor, and fair, respectively, the acceptability of these values in practice depends on a number of factors, including (i) improvement in prediction provided by the model over current practice and (ii) the cost–benefit analysis of correct versus false positive identification. In regards to EDs, we know that mental health literacy (Mond, 2014), detection and treatment access (Hart et al., 2011), and screening practice is extremely low. Furthermore, although screening tools can detect *current* EDs (Hill et al., 2010; Maguen et al., 2018), their utility in predicting *future* outcomes is unclear. If, by way of example, a screening tool based on the model of onset in the present study was to be implemented in a school setting (e.g., an online questionnaire with just the predictors of importance, linked to the model algorithm to determine risk score), it would have the potential to correctly discriminate between 75% of adolescents who will go on to develop an ED in the next 12 months—a significant improvement upon current school screening practices.

Psychological distress was found to be the most influential predictor within the ED onset model. Distress may be indicative of general lack of psychological wellbeing or the presence of psychiatric illness, including disorders other than EDs. Univariate analyses demonstrated that for every 5 points scored higher on a measure of psychological distress (the K-10, scoring range 0–50), the risk for an individual developing a probable ED within the next year increased by 75%. The role of distress in predicting ED onset is in line with the well-established evidence of distress (American Psychiatric Association, 2013; Hay & Williams, 2013; Mitchison et al., 2015; Stice, 2002) and psychiatric comorbidity (Ahn et al., 2019; Singleton et al., 2019) as transdiagnostic risk factors and correlates across many psychiatric disorders, include EDs.

On the other hand, ED-specific risk factors had variable importance in the models developed. Whereas weight and shape concerns were found to be important in the model for ED onset, weight loss dieting and weight status were not. These findings are at odds with the body of literature focusing on dieting and higher weight as risk factors for development of an ED, and targets for ED prevention programs. The current findings, however, do align with major EDetiological theory which posits that weight and shape concerns emerge prior to the development of ED behaviors, which subsequently further entrench weight and shape concerns, resulting in a vicious cycle (e.g., cognitive behavioral theory; Fairburn et al., 2003). Thus, focusing on cognitive variables (e.g., distress and weight and shape concerns) in screening, may enable the casting of an adequately “broad net” to pick up the majority of those who will go on to develop an ED before that event actually occurs. On the other hand, it should be acknowledged that due to our goal for model parsimony we included only one item assessing dieting. This single item may not have tapped into the construct of “risky dieting” as well as a full scale that has greater scope to capture the multi-dimensionality of this behavior. In regards to weight status not contributing significantly to onset prediction, this may reflect that EDs in the current sample were associated with the full spectrum of weight status from very low to very high weights (Mitchison et al., 2019), and underscores the need to focus less on absolute weight, (as opposed to weight *change*, which may be associated with disordered eating) and cognitive preoccupation with weight, when screening for ED risk.

This study found little discernible difference in the models developed using a traditional logistic regression approach as opposed to ML. This is similar to previous prediction research, including within the field of EDs predicting response to treatment (Espel-Huynh et al., 2021) and ED caseness (Krug et al., 2021). These findings suggest that, for many purposes, simpler analytic methods may be acceptable for interrogating questions pertaining to outcome prediction. On the other hand, studies with larger numbers of predictor variables have found an advantage to ML approaches in terms of accuracy (Haynos, Wang et al., 2021; Sadeh-Sharvit et al., 2020) and reducing model factors to achieve parsimony (Krug et al., 2021). It should be noted that this study applied only one ML approach (elastic net). Researchers in the ED field have started to address the question of which ML approach works best under which conditions (Krug et al., 2021), which should lead to greater clarity for statistical planning in future.

### 4.1 | Strengths and limitations

A strength of this study is the application of prognostic modeling methodology (Steyerberg et al., 2013), including the use of ML as an analytical tool. We used a prospective design, which counters problems with retrospective recall (e.g., Krug et al., 2021), and selected only predictors with known prognostic value, to balance model accuracy with parsimony. This pilot research also benefited from a large demographically diverse and phenotypically-rich community sample of adolescents with outcome measures inclusive of the full spectrum of EDs.

A primary limitation of this study was the absence of several predictors which could have improved model performance. The present study relied on secondary analyses of existing data, which precluded preselection of the full suite of evidence-based risk factors (Steyerberg et al., 2013) for ED onset (e.g., duration of illness, perfectionism, and psychiatric comorbidity) or ED persistence (e.g., perfectionism, perceived pressure to be thin, and age at puberty/menarche for ED onset) (Bakalar et al., 2015; Culbert et al., 2015; Day, Bussey, Trompeter, & Mitchison, 2021; de Portela Santana et al., 2012; Ghaderi, 2001; Glashouwer et al., 2019; Jacobi et al., 2004; Keel & Forney, 2013; Lie et al., 2019; Mazzeo & Bulik, 2009; Micali, 2005; Stice, 2002; Vall & Wade, 2015). Other limitations of the current study include lack of preregistration, the relatively small sample size compared to other risk model development studies, which precluded investigation into diagnostic and gender spectrum differences; the use of EDE-Q data for both predictor and outcome information, which may have artificially inflated the strength of observed relationships; and other limitations noted in previous studies using these data, including self-report and single-item measurement of some variables, and measurement of some of the diagnostic criteria over a 1 month as opposed to the DSM-5 3 month time period. Of note, the models developed in this study used population-based data and are only generalizable to community-based settings. Further, while the model as described in this study for ED persistence, once improved, will be useful in guiding decisions about *whether* to intervene, it cannot guide clinicians on the type of treatments that may ultimately be beneficial. A model that can guide treatment decisions in this way would also be of value, and should be a goal of future research, making use of evidence-based predictors of treatment moderators.

### 4.2 | Clinical and public health implications and future research

The finding that prediction of outcomes worked best when considering both transdiagnostic and ED-specific predictors emphasizes the need to move away from a focus on single risk factors in ED prediction, screening and interventions, and rather to consider multivariable approaches capitalizing on best known risk factors, whether they be disorder-specific or transdiagnostic. Of note, current screening instruments have tended to be ED specific (Hill et al., 2010; Maguen et al., 2018). The aim at the heart of clinical risk prediction is to translate evidence into practice by producing prediction tools. These for instance may be developed in the form of online risk calculators, such as the well-known Framingham Risk Scores for heart disease, that can be easily used by consumers and clinicians to guide shared decision-making about if, when and how to commence intervention. Such instruments are readily available for a variety of medical and psychiatric (Fusar-Poli et al., 2019) outcomes but are not yet developed for EDs. We recommend that researchers seeking to develop such prediction tools follow established prognostic modeling guidelines (Hemingway et al., 2013; Hingorani et al., 2013; Riley et al., 2013; Steyerberg et al., 2013). This involves producing highly accurate models using large cohort data that is inclusive of all relevant evidence-based risk factors. The next step prior to implementation of the risk model is external validation (and re-calibration if needed) in new cohorts which will define the parameters of the generalizability of the prediction model, including along dimensions of gender, age, and ethnicity/race. According to systematic reviews, external validation is an often overlooked step (Schmidt et al., 2017), limiting the utility of many models. The final step is impact testing which involves the transformation of statistical models into online calculators and testing their uptake and effectiveness in real world settings.

## 5 | CONCLUSIONS

This study has provided further evidence of the feasibility of developing risk prediction models for ED outcomes and developed the first models in a general population-based mixed gender sample of adolescents. The models performed relatively well considering the limitations of pre-existing data, giving confidence in future modeling work. Researchers are encouraged to continue this work, with eventual translation of evidence-based models to improve prevention and treatment for youth with EDs in mind.

## Supplementary Material

Supporting information file 1

Supporting information file 2

Supporting information file 3

## Figures and Tables

**TABLE 1:** Demographic characteristics and scores on predictors at Wave 1 for participants used for Aim 1 (predicting eating disorder onset) and Aim 2 (predicting eating disorder persistence).

	Eating disorder onset sample (Aim 1), *N* = 687		Eating disorder persistence sample (Aim 2), *N* = 276	
	*n* (%)			
Onset of eating disorder after 1 year	16.9		n/a	
Persistence of eating disorder at 1 year	n/a		74.6	
Sex^a^				
Male	401 (58.4)		54 (24.5)	
Female	286 (41.6)		166 (75.5)	
Country of birth				
Australia	577 (84.0)		232 (84.1)	
Other	110 (16.0)		44 (15.9)	
Bullying history	257 (41.9)		149 (64.5)	
		**Mean (*SD*)**		
Age		14.4 (3.6)		15.0 (1.5)
BMI percentile		47.2 (30.3)		62.0 (31.0)
Strict weight loss dieting days past month		0.5 (2.4)		4.3 (7.8)
Psychological distress		14.5 (5.8)		30.5 (10.3)
Weight and shape concerns		0.8 (1.0)		3.7 (1.7)
Purging episodes past month				
Self-induced vomiting		0.1 (0.7)		1.3 (4.4)
Laxatives		0.1 (1.0)		0.8 (4.3)
Social functioning		91.0 (15.5)		71.3 (26.6)

a Includes responses to “what is your gender: male, female” for one school who did not want to include separate questions on biological sex and gender.

## Data Availability

The data that support the findings of this study are available on request from the corresponding author. The data are not publicly available due to privacy or ethical restrictions.
