# Supplementary material for: Development of transdiagnostic clinical risk prediction models for 12-month onset and course of eating disorders among adolescents in the community
Source: Int J Eat Disord. Author manuscript; Available in PMC 2024 Jul 1. (PMC10404110; doi:10.1002/eat.23951)
Supplement: Supporting information file 1 [file NIHMS1910568-supplement-Supporting_information_file_1.docx]

**SUPPLEMENTARY FILE 1: MODEL PERFORMANCE METRICS EXPLAINER**

Below is a definition for each of the model performance metrics referred to in this study. For more information on each of these metrics and their interpretation, we refer readers to:

Wang SB. (2021). Machine learning to advance the prediction, prevention, and treatment of eating disorders. *European Eating Disorders Review, 29*, 683-691.

**Accuracy**

The proportion of all cases that are accurately detected by the model as either true positive or true negative cases. Bound by 0 to 1, with higher scores indicating better model performance.

**Positive predictive value (PPV)**

The proportion of all the cases identified by the model as positive which are true positive cases. Bound by 0 to 1, with higher scores indicating better model performance.

**Sensitivity (i.e., True Positive Rate)**

The proportion of all the actual positive cases that are detected by the model as a true positive case. Bound by 0 to 1, with higher scores indicating better model performance.

**Specificity (i.e., True Negative Rate)**

The proportion of all the actual negative cases that are detected by the model as a true negative case. Bound by 0 to 1, with higher scores indicating better model performance.

**Area under the receiver operating characteristic curve (AUC)**

Measures area under a curve with 1 – specificity on the x-axis and sensitivity on the y-axis. Bound by 0 to 1, with higher scores indicating better model performance. An AUC of 0.5 indicates chance-level predictive accuracy and an AUC of 1.0 indicates perfect classification (approximate guidelines for interpretation are as follows: 50 - .59 = extremely poor; .60 - .69 = poor; .70 - .79 = fair; .80 - .89 = good; .90 -1.00 = excellent).

**Area under the precision-recall curve (AUPRC)**

Measures area under a curve with sensitivity on the x-axis and positive predictive value on the y-axis, and is relative to the proportion of positive cases. Indicates the extent to which the model correctly identifies as many positive cases as it can without too many false positives. Bound by 0 to 1, with higher scores indicating better model performance.

**Brier score**

Measures the error of probabilistic predictions from binary outcomes. While there is no set value for a “good” Brier score, values can be compared between models. Bound by 0 to 1, with lower scores indicating better model performance.
