# Supplementary material for: Development of transdiagnostic clinical risk prediction models for 12-month onset and course of eating disorders among adolescents in the community
Source: Int J Eat Disord. Author manuscript; Available in PMC 2024 Jul 1. (PMC10404110; doi:10.1002/eat.23951)
Supplement: Supporting information file 2 [file NIHMS1910568-supplement-Supporting_information_file_2.docx]

**SUPPLEMENTARY FILE 2: OPERATIONALISATION OF EATING DISORDER DIAGNOSIS**

Text and Table below adapted from:

Mitchison D, Mond J, Bussey K, Griffiths S, Trompeter N, Lonergan A, Pike KM, Murray SB, Hay P (2019). DSM-5 full syndrome, other specified, and unspecified eating disorders in Australian adolescents: prevalence and clinical significance. *Psychological Medicine* 1–10. https://doi.org/10.1017/S0033291719000898

***Eating disorder diagnoses***

Supplementary Table 2 provides the operationalization of the diagnostic criteria. Most symptoms were captured by items of the Eating Disorder Examination Questionnaire (EDE-Q), which assesses the presence and severity of cognitive and behavioral eating disorder symptoms and features (Fairburn and Beglin, 2008). This questionnaire has previously been validated in Australian adolescents boys and girls and demonstrates sound reliability (Mond et al., 2014). Items used in this study included the behavioral frequency items (self-induced vomiting, laxative misuse, driven exercise, and binge eating), and the Likert-type items that comprise the combined weight and shape concern subscales. As the frequency of behaviors was only assessed over the past one month (not the three months duration required for BN and BED), we use the term ‘probable’ for these diagnoses. McDonald’s omega for the combined weight and shape concern subscale in the present study was 0.96 and 0.94 for girls and boys, respectively.

Participants self-reported current weight and height, which was converted to age and gender-adjusted body mass index (BMI) percentiles for children and adolescents. A BMI percentile <10 was used for the underweight criterion of AN, as this cut-off has most frequently been used in adolescent epidemiological studies of DSM-5 AN (Allen et al., 2013; Rojo-Moreno et al., 2015; Hammerle et al., 2016; Nagl et al., 2016). Three items from the Night Eating Questionnaire (NEQ) (Allison et al., 2008) were used to assess symptoms of NES, including a proportion of daily food intake consumed following supper, nocturnal eating (eating after going to bed), and awareness during nocturnal eating. The NEQ has been validated in adolescents and is superior to parent report (Gallant et al., 2012a).

Several additional questions were developed by the researchers to capture frequency of additional extreme weight control behaviors (fasting, strict dieting, detoxes, insulin misuse, other drug use for weight loss), distress associated with binge eating, and additional diagnostic BED features (e.g. eating faster than usual, eating alone due to embarrassment). Participants were also asked about any recent weight loss in the past 4 weeks to assess AAN.

Scores from the K10 Psychological Distress Scale (K10) (Kessler et al., 2002) and the Pediatric Quality of Life Scale (PedsQL) SF15 (Varni et al., 2001; Varni et al., 2003) were used to measure clinically significant distress and functional impairment, respectively. The K10 measures the frequency of anxiety and depressive symptoms during the past 4 weeks using 10 Likert-type items. Scores range from 10 to 50, with higher scores indicating higher levels of distress. The K‐10 has demonstrated high internal consistency and validity in predicting clinically significant levels of distress in general population samples (Kessler et al., 2002). McDonald’s omegas for the K-10 in girls and boys in the present study were 0.94 and 0.93, respectively. The 12 items from the physical functioning, emotional functioning, and social functioning subscales of the PedsQL SF15 (Varni et al., 2001, 2003) were included in the survey. Items ask participants to indicate on a Likert type scale how true a series of statements are of them in the past 4 weeks. Scores are reversed and transformed on a 0–100 scale, such that higher scores indicate higher functioning. Subscale scores are derived as the mean of the items for that scale. For this study we combined the emotional and social functioning scales to create a psychosocial subscale. The PedsQL SF15 has evidence of good reliability and validity in previous studies of adolescents (Varni et al., 2003). McDonald’s omegas in the current study sample for the physical functioning subscale was 0.86 and 0.87 for girls and boys respectively, and for the psychosocial functioning subscale was 0.90 and 0.91 for girls and boys respectively. Cut off scores of K-10 score >15 (indicative of moderate to severe distress) and/or PedsQL (physical or psychosocial subscale score) ⩽1 S.D w. below the sample mean were used. These K-10 cut-offs have been used previously in population-based studies (Andrews and Slade, 2001; Varni et al., 2003), and the PedsQL cut-off is more conservative than cut-offs used previously to identify children with special health care needs and chronic conditions (Huang et al., 2009).”

***Table S2. Operationalisation of DSM-5 Eating Disorder Diagnoses***

| Diagnosis | Study Criteria |
| --- | --- |
| **Criterial Eating Disorders** | |
| Anorexia nervosa (AN) | Current BMI percentile < 10; AND persistent extreme weight control behavior (fasting/strict dieting/detox, self-induced vomiting, laxative misuse, driven exercise, or misuse of insulin or other drugs) OR fear of weight gain OR felt fat over the past 4 weeks; AND extreme weight/shape concerns over the past 4 weeks |
| Probable bulimia nervosa (BN) | At least 4 objective binge eating episodes in past 4 weeks; AND persistent extreme weight control behavior in the past 4 weeks (fasting/strict dieting/detox, self-induced vomiting, laxative misuse, driven exercise, or misuse of insulin or other drugs); AND overvaluation of weight and/or shape over the past 4 weeks; AND not meeting criteria for AN |
| Probable binge eating disorder (BED) | At least 4 objective binge eating episodes in past 4 weeks; AND binge eating associated with 3 or more features (rapid eating, eating until uncomfortably full, non-hungry eating, eating alone, feeling disgusted/guilty/depressed after eating); AND marked distress regarding the binge eating; AND absence of persistent extreme weight control behavior over the past 4 weeks (fasting/strict dieting/detox, self-induced vomiting, laxative misuse, driven exercise, or misuse of insulin or other drugs); AND not meeting criteria for AN or BN |
| **Other Specified Feeding and Eating Disorder (OSFED)** | |
| Atypical anorexia nervosa (AAN) | Current BMI percentile > 10; AND lost weight in the past 4 weeks; AND persistent extreme weight control behavior (fasting/strict dieting/detox, self-induced vomiting, laxative misuse, driven exercise, or misuse of insulin or other drugs) OR fear of weight gain OR felt fat over the past 4 weeks; AND extreme weight/shape concerns over the past 4 weeks; AND not meeting criteria for AN or BN or BED |
| Subthreshold bulimia nervosa (SBN) | At least 2 objective binge eating episodes in past 4 weeks; AND at least 2 episodes of extreme weight control behavior in the past 4 weeks (fasting/strict dieting/detox, self-induced vomiting, laxative misuse, driven exercise, or misuse of insulin or other drugs); AND overvaluation of weight and/or shape over the past 4 weeks; AND not meeting criteria for AN or BN or BED |
| Subthreshold binge eating disorder (SBED) | At least 2 objective binge eating episodes in past 4 weeks; AND binge eating associated with 3 or more features (rapid eating, eating until uncomfortably full, non-hungry eating, eating alone, feeling disgusted/guilty/depressed after eating); AND marked distress regarding the binge eating; AND absence of persistent extreme weight control behavior over the past 4 weeks (fasting/strict dieting/detox, self-induced vomiting, laxative misuse, driven exercise, or misuse of insulin or other drugs); AND not meeting criteria for AN or BN or BED |
| Purging disorder (PD) | No binge eating in the past 4 weeks; AND at least 4 episodes of purging in the past 4 weeks (self-induced vomiting, laxative misuse, detox)†; AND not meeting criteria for AN or BN or BED |
| Night eating syndrome (NES) | Night time wakening and eating with awareness at least once/week OR consumption of the majority of daily intake following supper; AND significant psychological distress OR significant functional impairment; AND not meeting criteria for AN or BN or BED |
| **Unspecified Feeding or Eating Disorder (UFED)** | |
| UFED | Persistent binge eating or extreme weight loss behaviour in the past 4 weeks; AND extreme weight/shape concerns over the past 4 weeks; AND significant psychological distress OR significant functional impairment; AND not meeting criteria for AN or BN or BED or OSFED |
| † Frequency of purging based on the purging disorder criteria suggested by Keel and Striegel-Moore (2009) | |

***S2 References***

Allen KL, Byrne SM, Oddy WH and Crosby RD (2013) DSM-IV-TR and DSM-5 eating disorders in adolescents: Prevalence, stability, and psychosocial correlates in a population-based sample of male and female adolescents. Journal of Abnormal Psychology 122, 720–732.

Allison KC, Lundgren JD, O’reardon JP, Martino NS, Sarwer DB, Wadden TA, Crosby RD, Engel SG and Stunkard AJ (2008) The Night Eating Questionnaire (NEQ): psychometric properties of a measure of severity of the Night Eating Syndrome. Eating Behaviors 9, 62–72.

Fairburn CG and Beglin SJ (2008) Eating disorder examination questionnaire (EDE-Q 6.0). In Fairburn CG (ed.), Cognitive Behavior Therapy and Eating Disorders. New York: Guilford Press, pp. 309–313.

Gallant AR, Lundgren J, Allison K, Stunkard AJ, Lambert M, O’loughlin J, Lemieux S, Tremblay A and Drapeau V (2012a) Validity of the night eating questionnaire in children. International Journal of Eating Disorders 45, 861–865.

Hammerle F, Huss M, Ernst V and Bürger A (2016) Thinking dimensional: prevalence of DSM-5 early adolescent full syndrome, partial and subthreshold eating disorders in a cross-sectional survey in German schools. BMJ Open 6, e010843.

Huang I-C, Thompson LA, Chi Y-Y, Knapp CA, Revicki DA, Seid M and Shenkman EA (2009) The linkage between pediatric quality of life and health conditions: Establishing clinically meaningful cutoff scores for the PedsQL. Value in Health 12, 773–781.

Kessler RC, Andrews G, Colpe LJ, Hiripi E, Mroczek DK, Normand S-L, Walters EE and Zaslavsky AM (2002) Short screening scales to monitor population prevalences and trends in non-specific psychological distress. Psychological Medicine 32, 959–976.

Mond J, Hall A, Bentley C, Harrison C, Gratwick-Sarll K and Lewis V (2014) Eating-disordered behavior in adolescent boys: eating disorder examination questionnaire norms. International Journal of Eating Disorders 47, 335–341.

Nagl M, Jacobi C, Paul M, Beesdo-Baum K, Hofler M, Lieb R and Wittchen HU (2016) Prevalence, incidence, and natural course of anorexia and bulimia nervosa among adolescents and young adults. European Child & Adolescent Psychiatry 25, 903–918.

Rojo-Moreno L, Arribas P, Plumed J, Gimeno N, Garcia-Blanco A,Vaz-Leal F, Luisa Vila M and Livianos L (2015) Prevalence and comorbidity of eating disorders among a community sample of adolescents: 2-year follow-up. Psychiatry Research 227, 52–57.

Varni JW, Seid M and Kurtin PS (2001) PedsQL 4.0: reliability and validity of the Pediatric Quality of Life Inventory version 4.0 generic core scales in healthy and patient populations. Medical Care 39, 800–812.

Varni JW, Burwinkle TM, Seid M and Skarr D (2003) The PedsQL 4.0 as a pediatric population health measure: Feasibility, reliability, and validity. Ambulatory Pediatrics 3, 329–341.
