# Supplementary material for: Development of transdiagnostic clinical risk prediction models for 12-month onset and course of eating disorders among adolescents in the community
Source: Int J Eat Disord. Author manuscript; Available in PMC 2024 Jul 1. (PMC10404110; doi:10.1002/eat.23951)
Supplement: Supporting information file 3 [file NIHMS1910568-supplement-Supporting_information_file_3.docx]

**SUPPLEMENTARY FILE 3: UNIVARIATE ANALYSES**

Univariate logistic regressions were conducted to examine the unadjusted relationships between each candidate predictor and the outcomes. These univariate analyses were used to describe the relationships between variables in the models, and did not influence predictor selection, which was based on a priori evidence. A two-sided *p-*value < .05 was considered statistically significant. Table S3 presents the univariate relationships between each candidate predictor and the outcomes.

*Aim 1: Eating disorder onset.* Female adolescents were about 3-times more likely than male adolescents, and bully victims were almost 2-times more likely than non-victims, to have experienced onset of a new probable eating disorder by Wave 2. Psychological distress and weight and shape concerns were on average in the mild range among adolescents who experienced onset of a probable eating disorder by Wave 2. In contrast, adolescents who did not develop a probable eating disorder by Wave 2, on average reported negligible psychological distress and weight and shape concerns one year earlier. Very strict weight loss dieting and BMI percentile at Wave 2 were not statistically significant univariate predictors of probable eating disorder onset one year later.

Table S3. *Univariate relationships between individual candidate predictors and the outcomes of eating disorder onset (Aim 1) and eating disorder persistence (Aim 2)*

|  | **Eating disorder onset**  **(*n* =116)** | **No eating disorder**  **(*n* = 571)** | **OR (95% CI)** | ***p*** |
| --- | --- | --- | --- | --- |
| *Categorical Predictors, n (%)* |  |  |  |  |
| Sex  Male  Female | 42 (10.5)  74 (25.9) | 359 (89.5)  212 (74.1) | 2.98 (1.57, 4.52) | < .001 |
| Bully victim  Yes  No | 50 (19.5)  42 (11.8) | 207 (80.5)  314 (88.2) | 1.81 (1.16, 2.82) | .009 |
| *Continuous Predictors, M (SD)* |  |  |  |  |
| Psychological distress | 19.25 (9.07) | 13.51 (4.28) | 1.15 (1.11, 1.18) | < .001 |
| Weight and shape concerns | 2.43 (1.24) | 1.62 (0.86) | 2.01 (1.67, 2.42) | < .001 |
| Very strict weight loss dieting days | 0.62 (2.20) | 0.49 (2.49) | 1.02 (0.95, 1.10) | .593 |
| BMI percentile | 47.95 (29.12) | 47.00 (30.51) | 1.00 (0.99, 1.01) | .761 |
|  |  |  |  |  |
|  | **Persistent eating disorder (*n* = 206)** | **Remitted eating disorder**  **(*n* = 70)** | **OR (95% CI)** | ***p*** |
| *Continuous Predictors, M (SD)* |  |  |  |  |
| Psychological distress | 31.77 (9.72) | 26.50 (10.99) | 1.06 (1.03, 1.09) | < .001 |
| Self-induced vomiting (episodes past month) | 1.52 (4.82) | 0.59 (2.57) | 1.09 (0.99, 1.22) | .161 |
| Laxative use (episodes past month) | 0.99 (4.89) | 0.36 (1.69) | 1.06 (.95, 1.18) | .328 |
| Social functioning | 69.35 (26.84) | 77.08 (25.24) | 0.99 (0.98, < 1.00) | .040 |

*Aim 2: Eating disorder persistence.* The strongest Wave 1 univariate predictors of Wave 2 eating disorder persistence were psychological distress and social functioning, with scores on distress being “high” on average among adolescents who were identified as remitted from their probable eating disorder after one year, but “very high” for adolescents who were identified as having a persistent probable eating disorder. Likewise, although social functioning was impaired for both groups with a probable eating disorder at Wave 1, the impairment was greater among those who were classified as having a persistent probable eating disorder one year later versus those who were classified as remitted. Neither forms of purging (self-induced vomiting or laxative use) were statistically significant univariate predictors of the persistence of a probable eating disorder after one year.
